# Randomized trial to evaluate contraceptive efficacy, safety and acceptability of a two-rod contraceptive implant over 4 years in the Dominican Republic^☆^

**DOI:** 10.1016/j.conx.2019.100006

**Published:** 2019-03-18

**Authors:** M.J. Steiner, V. Brache, D. Taylor, R. Callahan, V. Halpern, A. Jorge, S. Wevill, J. Sergison, L. Venkatasubramanian, L. Dorflinger

**Affiliations:** a Contraceptive Technology Innovation Division, FHI 360, 359 Blackwell Street, Durham, NC 27701, USA; b Asociación Dominicana Pro Bienestar de la Familia, Inc. (PROFAMILIA), Santo Domingo, Dominican Republic

**Keywords:** Sino-implant, Levoplant, Long-acting reversible contraceptive, Contraceptive effectiveness, safety

## Abstract

**Objective:**

Sino-implant (II) is a contraceptive implant that had a commodity price one-third of the competing products a decade ago. To make Sino-implant (II) more widely available, we conducted a trial to collect safety and efficacy data required for World Health Organization (WHO) prequalification, a quality standard allowing global donors to procure a pharmaceutical product.

**Study design:**

This was a randomized controlled trial allocating 650 participants to either Sino-implant (II) or Jadelle®. Participants were seen at 1 and 6 months, and then semiannually. The primary efficacy measure was the pregnancy Pearl Index [number of pregnancies per 100 women-years (WY) of follow-up] in the Sino-implant (II) group during up to 4 years of implant use.

**Results:**

For the primary outcome, Sino-implant (II) had a 4-year Pearl Index of 0.74 (95% confidence interval, 0.36–1.37) compared to 0.00 (95% confidence interval, 0.00–1.04) for Jadelle®. The Sino-implant (II) pregnancy rate was significantly higher in the fourth year (3.54 per 100 WY) than in the first 3 years combined (0.18 per 100 WY; p <.001). Total levonorgestrel concentrations were equivalent between groups at month 12, but were 19%, 22% and 32% lower in the Sino-implant (II) group at months 24, 36 and 48, respectively (p <.001 at each time point). Safety and acceptability of the two products were similar, while providers documented significantly higher breakage rates during removal of Sino-implant (II) (16.3% vs. 3.1%; p <.001).

**Conclusion:**

Based on these results, WHO prequalified Sino-Implant (II) with a 3-year use label in June 2017, 2 years shorter than the 5-year duration of Jadelle®.

**Implications:**

WHO prequalification allows global donors to procure Sino-implant (II), which means women in many low resource countries will have greater access to highly effective and acceptable contraceptive implants. Our study noted important clinical differences, including shorter duration of high effectiveness with Sino-implant (II) when compared to the other available two-rod system, Jadelle®. Introduction strategies should include appropriate training on these differences.

## 1 Introduction

Sino-implant (II) is a subdermal contraceptive implant system manufactured in China and marketed globally as Levoplant™. To make Sino-implant (II) more broadly available to women in developing countries, the Bill & Melinda Gates Foundation funded a global initiative coordinated by FHI 360. A key objective of the initiative was to obtain World Health Organization (WHO) prequalification, which is necessary for global procurement agencies (e.g., United Nations Population Fund and US Agency for International Development) to distribute the product.

The initial Sino-implant (II) dossier was submitted to the WHO Prequalification Team: medicines (PQTm) in 2010 with data collected in China in the early 1990s and reviewed by Steiner et al [1]. WHO PQTm concluded that available data were insufficient to warrant prequalification, as the trials did not meet Good Clinical Practice (GCP) guidelines. FHI 360 subsequently undertook a GCP-compliant trial in the Dominican Republic (DR), with the main objective to evaluate the contraceptive efficacy of Sino-implant (II) during 4 years of use. Secondary objectives included comparing Sino-implant (II) safety, efficacy, acceptability and pharmacokinetics to Jadelle® during up to 5 years of use.

## 2 Methods

We conducted this phase III, randomized, active-control, parallel group clinical trial at the PROFAMILIA clinic in Santo Domingo, DR. The ethical review board at FHI 360 and two review boards in the DR (PROFAMILIA and CONABIOS) approved the protocol. We registered the trial on ClinicalTrials.gov and adhered to the CONSORT guidelines in our reporting of results [2].

The study had two treatment groups: Sino-implant (II) [Shanghai Dahua Pharmaceutical Co., Ltd. (Dahua)] and an active control, Jadelle® (Bayer Healthcare, Berlin, Germany). Each device consists of two flexible silicone rods loaded with 75 mg of levonorgestrel (LNG)–150 mg LNG per set. We randomized participants using sequentially numbered, sealed opaque envelopes. We instructed the clinicians to insert (and remove) the assigned contraceptive implant following instructions adapted from Jadelle®'s instructions [3].

To be eligible for the study, women had to be aged 18 to 44 years, not pregnant or lactating, and not wishing to become pregnant in the next 5 years (see Supplement for all inclusion/exclusion criteria). We enrolled eligible participants during the first 7 days of their menstrual cycle and confirmed negative pregnancy status per urine pregnancy test (Accu-Tell Rapid Diagnostic, HCG Urine/Serum Cassette; AccuBioTech Co., Ltd., Beijing, China; catalog no. ABT-FT-B2.). The Accu-Tell Rapid Diagnostic test detects hCG concentration of 25 mIU/ml and greater (sensitivity and specificity > 99.9%). The urine pregnancy test was repeated at the final visit and at any other visit where there were signs of pregnancy. A positive urine test was confirmed by ultrasound and/or serum quantitative hCG measurement.

We scheduled follow-up visits at 1, 6, 12, 18, 24, 30, 36, 42, 48, 51, 54, 57 and 60 months after insertion. We asked a subgroup of 50 participants to attend additional visits 6, 24, 48 and 72 h, and 7 and 90 days after insertion for LNG sampling to compare the initial pharmacokinetic (PK) profiles of the two products. At all regular visits, we measured blood pressure and weight, drew blood for determination of total LNG and sex hormone-binding globulin (SHBG) concentrations (only in final 150 enrolled participants, when funding became available for this extra testing), collected information on AEs and concomitant medication use and evaluated acceptability (at month 12, month 48 and final study visit).

We chose the study size of 650 women to meet criteria specified in the European Medicines Agency Guideline on Clinical Investigation of Steroid Contraceptives in Women [4]: specifically, 400 women completing 1 year of Sino-implant (II) use, 200 women completing the labeled 4-year duration of use in China and sufficient months of Sino-implant (II) use to obtain a two-sided 95% confidence interval (CI) for the pregnancy Pearl Index with a half-width ≤ 1%. Although the primary efficacy assessment of Sino-implant (II) was noncomparative, we included an active control to allow for a direct comparison of total LNG concentrations, safety and acceptability; the 4:1 allocation ratio was intended to provide sufficient precision for making such comparisons.

The primary efficacy measure was the pregnancy Pearl Index [number of pregnancies per 100 women-years (WY) of follow-up] in the Sino-implant (II) group during up to 4 years of implant use. Although our initial plans were to follow participants for up to 5 years as a secondary outcome (labeled duration of use of Jadelle®), on February 1, 2016, the independent data and safety monitoring board recommended participant follow-up be truncated at month 48 due to a higher-than-expected pregnancy rate among the women who had already provided data in the fourth and fifth years of Sino-implant (II) use. To assure that we detected any possible early pregnancies present at the 48-month visit, we decided to simultaneously test both urine and serum samples with the Accu-Tell Rapid Diagnostic test at this exit visit.

Secondary efficacy measures included cumulative probabilities of pregnancy and pregnancy rates at yearly intervals. We reported the pregnancy Pearl Index and pregnancy rates at yearly intervals with 95% CIs based on a Poisson assumption for mean time to event. We used Kaplan–Meier methods to estimate cumulative probabilities of pregnancy, with 95% CIs derived using the complementary log–log transformation. Although the study is not powered to detect differences in pregnancy risk between the two implants types, we compared the proportions of participants becoming pregnant based on an exact two-sided test.

PPD bio-analytical labs measured total plasma LNG concentrations using a validated high-performance liquid chromatography tandem mass spectrometry assay (interassay and intra-assay precision, expressed as the coefficient of variation times 100, ranged from 2.72% to 6.04% and from 1.60% to 9.00%, respectively) and serum SHBG using an ADVIA Centaur solid-phase two-site chemiluminescent immunoassay.

We reported *C*_max_ and *T*_max_ for each participant undergoing intensive PK sampling, excluding women with detectable LNG at baseline. We estimated corresponding AUC values using the linear-log trapezoidal method and summarized results by implant type using means, SDs, 95% CIs and other descriptive statistics. We compared groups using p values for tests of no difference and 90% CIs for geometric mean ratios of PK parameters. Although this was not a bioequivalence trial, for descriptive purposes, we considered the implant types equivalent with respect to a given PK parameter if the corresponding 90% CI fell in the interval (0.8–1.25) per standard guidance [5]. We summarized total LNG concentration among all enrolled, and SHBG and the free LNG index, defined as the ratio of total LNG (nmol/L) to SHBG (nmol/L) concentrations (times 100), in the final 150 enrolled participants, by study visit using descriptive statistics, with no adjustment for multiple comparisons.

For safety outcomes, we compared the percentage of women experiencing AEs within system organ class, the percentage experiencing complications during insertion or removal and the percentage of implants that broke during removal between groups using Fisher's Exact Tests.

For acceptability outcomes, we computed cumulative probabilities of early implant removal using Kaplan–Meier methods, with differences in rates assessed using a log-rank test. We compared categorical responses to acceptability questions between treatment groups using Fisher's Exact Tests. Unless otherwise noted, we conducted all tests at the two-sided α =.05 significance level, based on allocated treatment group.

## 3 Results

### 3.1 Study subjects

**Fig. 1:**
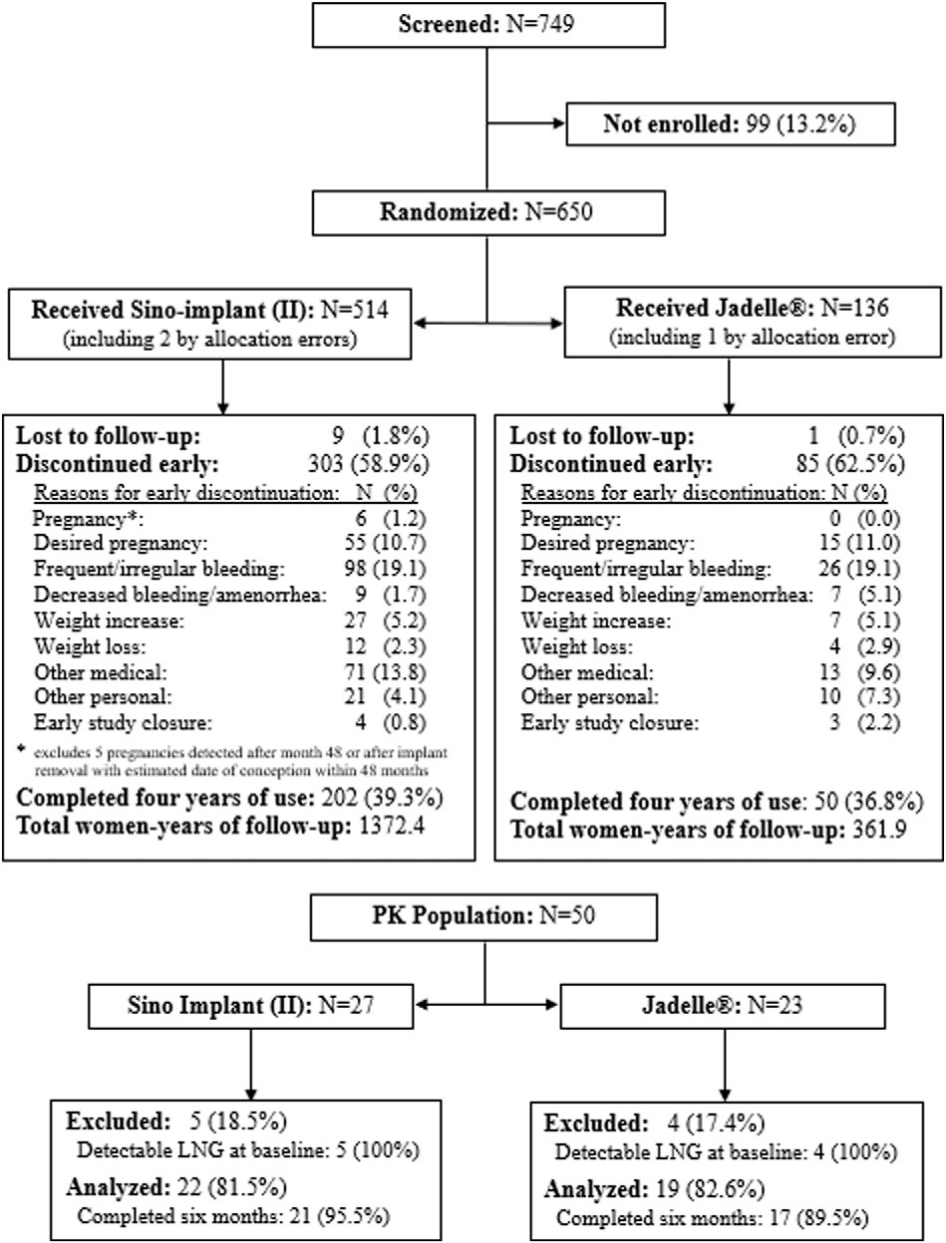
Participant flow diagram for a randomized control trial to evaluate the contraceptive efficacy, safety and acceptability of a two-rod contraceptive implant over 4 years in the DR.

We screened 749 women between October 2011 and July 2013 to randomize 650 participants into the trial that completed follow-up July 2017 (Fig. 1). Among the 650 enrolled participants, 514 received Sino-implant (II) and 136 received Jadelle® (including 3 random allocation errors discovered during the closeout monitoring visit). Only 10 participants were lost to follow-up, and the visit completion rate was greater than 95%. Baseline characteristics were well balanced across groups and are presented in Table 1.

**Table 1:** Baseline characteristics of women randomized to Sino-implant (II) or Jadelle® insertion

Variable	Sino-implant (II) (*n* = 514)	Jadelle® (*n* = 136)
Mean age (range), years	23.5 (18–39)	23.7 (18–36)
Race, *n* (%)		
Biracial	478 (93.0)	131 (96.3)
Black	20 (3.9)	2 (1.5)
White	16 (3.1)	3 (2.2)
Partner status, married or cohabitating, *n* (%)	362 (70.4)	106 (77.9)
Mean body mass index (range), kg/m^2^	24.7 (16–44)	24.4 (16–37)
Never pregnant, *n* (%)	18 (3.5)	1 (0.7)
Contraception last used, *n* (%)^a^		
Combined oral contraceptive pills	159 (30.9)	43 (31.6)
Progestin-only pill	8 (1.6)	2 (1.5)
Implant	14 (2.7)	3 (2.2)
IUD	6 (1.2)	4 (2.9)
Injectable	34 (6.6)	9 (6.5)
Condom	268 (52.1)	70 (51.5)
Other	20 (3.9)	4 (2.9)
Never used contraception	11 (2.1)	5 (3.7)
Regular menses, *n* (%)	486 (94.6)	131 (96.3)

a More than one response possible.

Of the 50 participants recruited into the PK population for more intensive assessment of total LNG concentrations (Fig. 1), we excluded 9 (18%) due to detectable LNG at baseline (range, 113–1860 pg/ml), leaving 41 participants [22 and 19 in the Sino-implant (II) and Jadelle® groups, respectively] contributing to the estimation of PK parameters. Baseline characteristic for this subgroup were well balanced and similar to the whole group, with the exception that women with body mass index ≥ 30 kg/m^2^ were excluded from the PK population (data not shown).

### 3.2 Efficacy

In the primary efficacy analysis, the 514 women assigned Sino-implant (II) contributed 1343.9 WY of implant use during up to 4 years of treatment, resulting in a 4-year Pearl Index of 0.74 (95% CI, 0.36–1.37; Table 3). We recorded 11 pregnancies in the study, all among the 514 women assigned to Sino-implant (II): 1, 1, 8 and 1 in years 2, 3, 4 and 5 of implant use, respectively. Of these 11 pregnancies, we recorded 2 ectopic pregnancies, 4 spontaneous including 2 chemical pregnancies and 1 induced abortion, and 4 live births including one set of twins without fetal or neonatal abnormalities (Table 2).

**Table 2:** Study pregnancies of women randomized to Sino-implant (II) insertion (no pregnancies recorded in the Jadell®e group)

PN	Months to EDF	Age (years)^a^	Weight (kg)^a^	LNG (pg/ml)^a^	LNG specimen^a^	Confirmed by ultrasound	Outcome
1002	40.2	28	65.8	135	Month 36	Yes	Ectopic pregnancy
1061	46.5	31	69.4	190	Month 42	Yes	Live birth
1158	48.5	33	78.5	122	Month 48	Yes	Induced abortion
1260	20.7	34	65.6	443	Month 18	Yes	Spontaneous abortion
1305	37.2	22	79.9	104	Month 36	Yes	Live birth
1347	47.9	26	82.8	103	Month 42	Yes	Live birth
1421	33.4	32	50.8	179	Month 30	Yes^b^	Ectopic pregnancy
1510	42.8	28	77.6	134	Month 42	Yes	Spontaneous abortion
1537	46.5	24	81.2	163	Month 42	No^c^	spontaneous abortion/chemical pregnancies
1556	36.9	26	78.3	182	Month 36	Yes	Live twins
1630	47.7	30	74.0	328	Month 42	No^d^	spontaneous abortion/chemical pregnancies

a Last measurement before EDF.

b Ultrasound identified a possible ectopic pregnancy in the left oviduct.

c Ambiguous urine test result, but serum hCG results were elevated and consistent with pregnancy. Repeated ultrasounds showed no evidence of pregnancy, and she reported spontaneous menses 16 days after study exit.

d Negative urine test, but a parallel qualitative hCG test was weakly positive and a quantitative hCG test was elevated (10.7 mIU/ml). She was positive by urine test 6 days later, but repeated ultrasounds showed no evidence of pregnancy and she reported spontaneous menses 12 days after study exit.

The 3-year Pearl Index based on 1117.7 WY was 0.18 (95% CI, 0.02–0.65). The corresponding yearly pregnancy rates increased slightly from 0.00 per 100 WY (95% CI, 0.00–0.79) in year 1 to 0.34 (95% CI, 0.01–1.92) in year 3, before rising to 3.54 per 100 WY (95% CI, 1.53–6.97) in year 4 (p <.001 in an exploratory test of no difference between rates in years 1 to 3 vs. year 4). In a sensitivity analysis that excluded the two chemical pregnancies, the pregnancy rate in year 4 declined to 2.65 per 100 WY (95% CI, 0.97–5.77).

The 136 participants assigned to Jadelle® contributed 353.2 WY of follow-up in the first 4 years of implant use. We recorded no pregnancies resulting in a Pearl Index of 0.00 (95% CI, 0.00–1.04; Table 3). The trial was not designed or sufficiently powered to compare the Pearl Indices between the two implant groups.

**Table 3:** Pearl Indices, by year of implant use and overall, of women randomized to Sino-implant (II) or Jadelle® insertion

Time period/group	Women^a^	WY of follow-up	Pregnancy events	Pearl Index (per 100 WY)	95% CI for Pearl Index
1st year of use					
Sino-implant (II)	514	465.6	0	0.00	(0.00–0.79)
Jadelle®	136	123.6	0	0.00	(0.00–2.98)
2nd year of use					
Sino-implant (II)	410	360.3	1	0.28	(0.01–1.55)
Jadelle®	109	98.3	0	0.00	(0.00–3.75)
3rd year of use					
Sino-implant (II)	323	291.8	1	0.34	(0.01–1.91)
Jadelle®	87	74.7	0	0.00	(0.00–4.94)
4th year of use					
Sino-implant (II)	259	226.2	8	3.54	(1.53–6.97)
Jadelle®	64	56.6	0	0.00	(0.00–6.52)
5th year of use^b^					
Sino-implant (II)	182	21.8	1	4.59	(0.12–25.6)
Jadelle®	48	5.3	0	0.00	(0.00–69.8)
Years 1–3, combined					
Sino-implant (II)	514	1117.7	2	0.18	(0.02–0.65)
Jadelle®	136	296.6	0	0.00	(0.00–1.24)
Years 1–4, combined^c^					
Sino-implant (II)	514	1343.9	10	0.74	(0.36–1.37)
Jadelle®	136	353.2	0	0.00	(0.00–1.04)
Years 1–5, combined^b^					
Sino-implant (II)	514	1365.7	11	0.81	(0.40–1.44)
Jadelle®	136	358.5	0	0.00	(0.00–1.03)

a Number of women on product at the start of each time period.

b Participants had already entered year 5 when data and safety monitoring board decision was made to truncate the study after year 4; only 5% of participants contributed > 3 months of implant use in year 5.

c Primary analysis result.

**Fig. 2:**
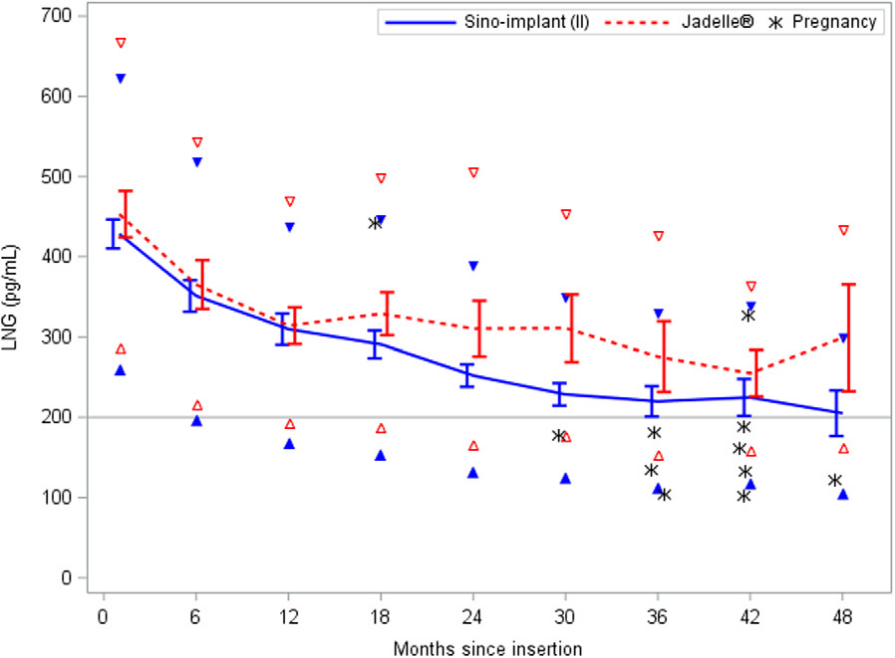
Arithmetic mean total LNG concentrations during 48 months of implant use in women randomized to Sino-implant (II) or Jadelle® insertion (95% CIs for means are shifted slightly for visibility). Solid and open triangles denote upper and lower 10th percentiles for Sino-implant (II) and Jadelle®. Asterisks are LNG concentrations at last time point before estimated date of fertilization among 11 pregnant women.

Sino-implant (II) users who became pregnant had a nonsignificantly higher mean body weight than the remaining users (73.1 kg vs. 66.0 kg: p =.09). In 9 of the 11 women who became pregnant (81.8%), the measured total LNG concentration at the last visit before EDF was below 200 pg/ml and was also below the average LNG concentration among all Sino-implant (II) users at the corresponding sampling visits (Table 2 and Fig. 2).

### 3.3 PK—total LNG concentrations

Total plasma LNG concentrations in the PK population uniformly exceeded 200 pg/ml within 24 h of implant insertion in both groups. The mean *C*_max_ values in the Sino-implant (II) and Jadelle® group, respectively, were 833 and 962 pg/ml; mean *T*_max_ values were 5.4 and 4.3 days; and mean AUC_0–6m_ values were 2489 and 2862 pg ∙ months/ml.

In the Sino-implant (II) group, mean concentrations decreased from 428 pg/ml 1 month after insertion to 310, 252, 220 and 205 pg/ml at months 12, 24, 36 and 48, respectively (Fig. 2). In the Jadelle® group, mean concentrations generally decreased from 453 pg/ml at month 1 to 314, 310, 276 and 299 pg/ml at months 12, 24, 36 and 48, respectively. The observed trend in decreasing geometric mean ratios over time was significant (p <.001) in an exploratory test of no difference in log-linear slopes [see detailed discussion of LNG levels, related SHBG levels and the free LNG Index (FLI) in Supplement].

### 3.4 Safety

Except for menstrual irregularities [experienced by 48.4% and 58.8% of Sino-implant (II) and Jadelle® users, respectively; p =.03], there were no significant differences in the proportions of women experiencing common AEs.

Twenty-eight participants (5.4%) in the Sino-implant (II) group reported a total of 32 SAEs, including 7 that were considered at least possibly related to implant use: 2 ectopic pregnancies, 2 ovarian cysts, 1 episode of cholecystitis, 1 episode of cholelithiasis and 1 case of biliary colic. Five participants (3.7%) in the Jadelle® group reported a total of six SAEs, none of which were considered related to implant use.

### 3.5 Implant insertion and removal

Implant insertion took an average of 32.7 (SD, 9.7) and 29.2 (SD, 8.8) s in the Sino-implant (II) and Jadelle® groups, respectively, and the insertion procedure was uniformly considered easy (100%) for both implant types by experienced clinicians. Most participants reported no pain during the insertion procedure [92.4% for Sino-implant (II) and 94.9% for Jadelle®; p =.35].

The implant removal took less than 5 min for 92.0% and 95.5% of Sino-implant (II) and Jadelle® procedures, respectively, although providers were less likely to report that Sino-implant (II) was easy to remove (82.4% and 93.2%; p <.01). Most participants reported no pain during the removal procedure [83.6% for Sino-implant (II) and 88.9% for Jadelle®; p =.14].

The total breakage rate during removal was significantly greater for Sino-implant (II) than for Jadelle® (16.3% vs. 3.1%; p <.001), and a second clinic visit was required to ensure that the Sino-implant (II) was completely removed in 13 (2.7%) instances. One of the identified explanations for the high breakage rate was that the site was not following the removal instructions and were applying twisting/torque motion instead of pulling when withdrawing the rods. Additional training of site clinicians reemphasized the instructions with respect to minimizing the use of twisting/torque when withdrawing the rods. Still later, the clinic began using less sharp and slightly larger Crile forceps instead of mosquito clamps for withdrawing the rods. The breakage rate in the Sino-implant (II) group generally decreased with each intervention: 33.3% prior to retraining, 17.6% after training to minimize twisting/torque and 8.3% after the site began using Crile forceps. However, the Sino-implant (II) breakage rate increased to 24.8% in the 3-month period following the decision to truncate follow-up (when the number of removals was greatest) and remained somewhat elevated thereafter (14.1%).

### 3.6 Acceptability

Year 4 continuation rates were similar for Sino-implant (II) and Jadelle® (41% vs. 38%; p =.69), with about 20% of participants in both groups discontinuing annually. The most common reasons for wanting the implant removed early were frequent or irregular bleeding, 19.1% in each group. Similar proportions of participants using Sino-implant (II) and Jadelle® said that they were very satisfied/satisfied with their assigned implant (85.4% and 83.7%, respectively), and most (96.8% and 95.6%, respectively) would recommend implants to a friend/relative.

## 4 Discussion

Results confirm that Sino-implant (II) is a highly effective, long-acting contraceptive method, with an estimated Pearl Index of 0.74 per 100 WY during up to 4 years of use and with safety and acceptability profiles that are similar to Jadelle®. The Sino-implant (II) pregnancy rate was significantly higher in the fourth year of use (3.54 per 100 WY) than in the first 3 years combined (0.18 per 100 WY). As a result, WHO prequalified the product with a 3-year use label [6]. Some, but not all, earlier Chinese trials found decreased contraceptive efficacy beyond year 3 [1], although none recorded as sharp a decrease as we saw in this trial in the DR. The supportive cohort study described in this issue [23] recorded four pregnancies (three during the third and one during the fourth year) resulting in a higher pregnancy rate during year 3 (1.34; 95% CI, 0.28–3.93) than year 4 (0.44; 95% CI, 0.01–2.47) or year 5 (0.00; 95% CI, 0.00–2.02).

What might explain these somewhat different results across studies? The trial in the DR was the more rigorous study from a design and implementation perspective (e.g., randomized; low loss-to-follow-up; site inspection per WHO GCP). How much of the difference in efficacy between studies in China and the DR is due to (1) differences in sexual behavior and other covariates related to the underlying risk of pregnancy (e.g. age), (2) ethnic/genetic differences in pharmacokinetics and pharmacodynamics of LNG, (3) random variability and inherent challenges of measuring pregnancy outcomes or (4) data quality (e.g., possibility of participants accessing abortions without site staff knowledge) is not known.

Sexual behavior is notoriously difficult to measure [7], and most modern contraceptive efficacy trials [8], [9], [10] make no attempts to control for this covariate because doing so might introduce additional confounding. Participants in the DR trial were on average younger than in the China study at enrollment (23.6 vs. 33.9 years old, respectively), which is perhaps associated with increased sexual frequency and somewhat higher fecundity [11]. Moreover, participants in the DR trial had somewhat higher body mass index than participants in the China study (24.6 vs. 23.7, respectively), which also has been shown to increase the risk of pregnancy in some, but not all, contraceptive implant trials [12]. Thus, it is possible that participants in the DR trial were exposed to higher underlying risk of pregnancy than women included in the most recent China study.

Differences in metabolism of hormones like LNG and MPA between Asian and non-Asian females as well as males are well documented [13], [14], [15]. LNG levels were generally higher in the China study than in the DR trial and did not show the same downward trend after year 3. However, the PK outcome related to the free drug concentration (FLI), presumed to be more highly correlated with pregnancy prevention than total LNG [16], [17], [18], was stable after year 3 in the DR (see PK Supplement) but declined in China. We must be careful not to overinterpret PK differences and temporal trends because these are nonrandomized comparisons.

Given the expected rarity of pregnancy in implant trials, the study was not powered to detect, nor did it identify, significant differences in pregnancy rates between implant types. However, we did observe a significantly higher pregnancy rate in the fourth year of Sino-implant (II) use than in the first 3 years combined. Of the eight pregnancies in the 4th year, one chemical pregnancy was only detected because of the deviation from the pregnancy testing algorithm specified in the protocol, and a second pregnancy was included in the analysis because we could not determine with certainty that the EDF was outside the follow-up period. This illustrates the challenge of conducting contraceptive trials with the inherent difficulty of dating conception as well as the fact that 30%–50% of pregnancies are not viable and end spontaneously in early pregnancy loss [19]. The latter can lead to substantially different efficacy outcomes depending on the frequency of pregnancy testing and the sensitivity of tests used. Finally, differences in data quality can never be ruled out for potentially explaining differences in efficacy.

We observed a higher than expected breakage rate for Sino-implant (II) at the time of removal. At the beginning of FHI 360's involvement with the manufacturer of Sino-implant (II), we conducted an assessment of the clinical experience with removal in China [20]. Among 318 removals we assessed, 16 (5.0%) implants broke, which is comparable to breakage rates for other contraceptive implants [21], [22] as well as the rate we found in the China study presented in this issue [23]. When we noted substantially higher breakage rates in the current trial, the study team explored the potential reasons and recommended procedures to improve the removal technique. While laboratory testing conducted during the trial showed that the tensile integrity of Sino-implant (II) was less robust than Jadelle®'s, the reduction in breakage after retraining suggests removal technique is an important factor that can lead to varying breakage rates across and within studies. That said, breakage rates increased again during study close-out when the number of procedures per day increased. The significantly higher breakage rate of Sino-implant (II) compared to Jadelle® (16.3% vs. 3.1%; p <.001) may be one of several factors (e.g., commodity price, duration of use, lead time for shipping, etc.) that country stakeholders and global procurement agencies will consider when deciding what type of implant to distribute.

Based on the results of the DR trial along with substantial manufacturing systems improvements, WHO has prequalified Sino-Implant (II) with a 3-year use label [6]. Given the long-standing 4-year approval in China as well as reassuring results of the supportive cohort study [23], Sino-implant (II) will likely remain a 4-year product in China. Similarly, some national drug regulatory authorities may assess the entirety of the clinical data and conclude that they support the marketing as a 4-year product. Clear instructions to both providers and clients will be necessary on the duration of use of this product, as well as the two other widely available contraceptive implants (Jadelle® and Nexplanon®) with their respective 5- and 3-year duration of use, to avoid confusion.

**Fig. 3:**
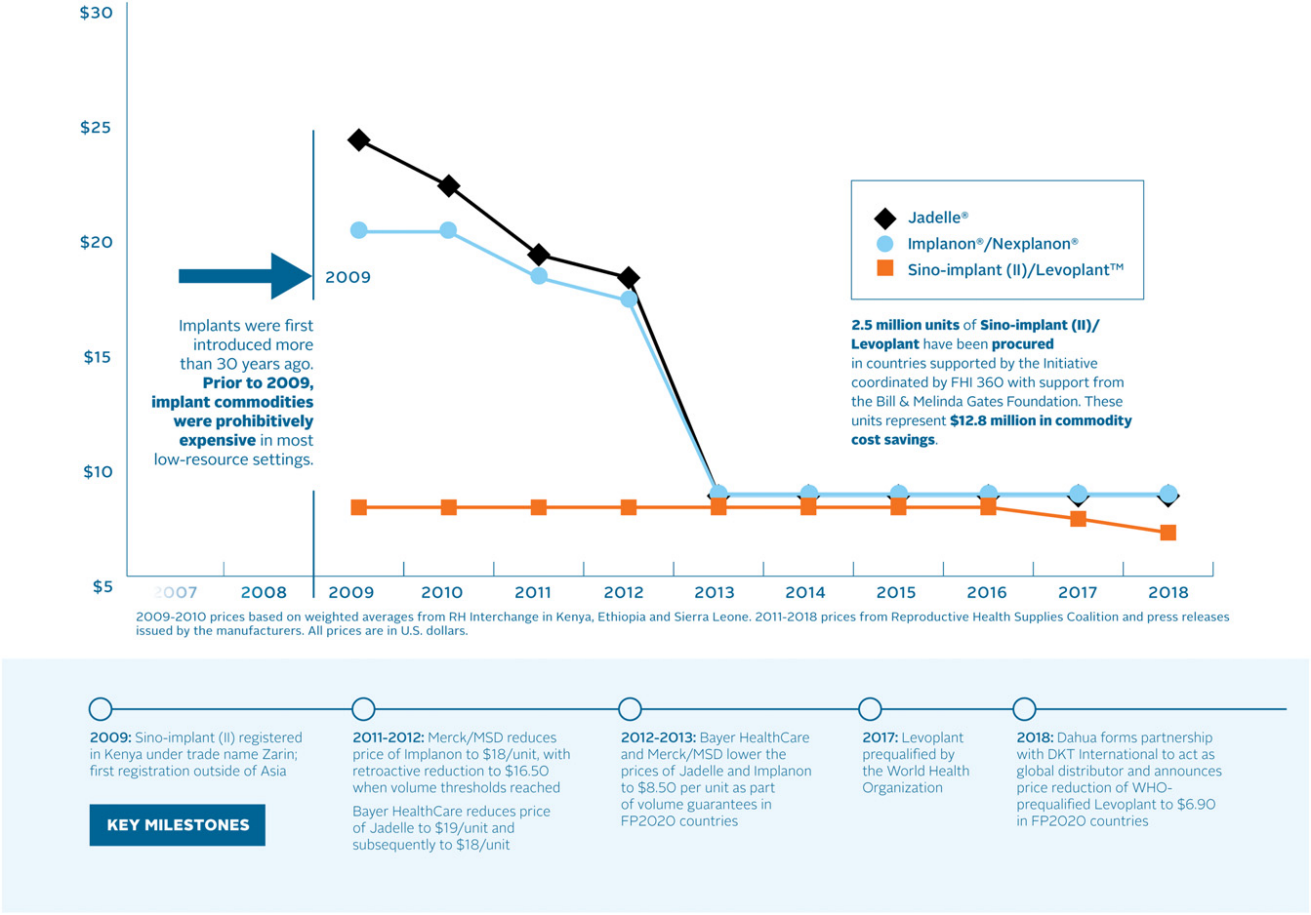
Market shaping through price competition.

While one key outcome of the Sino-implant (II) initiative was achieved with WHO prequalification in June 2017, a more important legacy is the catalytic role the product has played in bringing price competition to the market (Fig. 3)—helping women in low-resource countries have greater access to highly effective, acceptable and more affordable contraceptive implants.

## Appendix A Supplementary data

Supplementary material Image 1
