# Supplementary material for: Randomized trial to evaluate contraceptive efficacy, safety and acceptability of a two-rod contraceptive implant over 4 years in the Dominican Republic
Source: Contracept X. 2019 Mar 18;1:100006. doi: 10.1016/j.conx.2019.100006 (PMC7252426; doi:10.1016/j.conx.2019.100006)

Supplementary Material:

Complete Inclusion/Exclusion Criteria

To be eligible for the study, women had to be: in good general health; aged 18 to 44 years; not pregnant or lactating; not wishing to become pregnant in the next five years; requesting long-acting reversible contraception; > 9 months after last injection of Depo Provera, > 3 months after last injection of combined injectable contraceptive, > than 1 week after last intake of LNG-containing pill or implant removal; able to understand information about study participation; willing to sign consent form; and able to return for follow-up visits over five years. Exclusion criteria were: acute deep venous thrombosis and/or pulmonary embolism or history of thromboembolic disease; systemic lupus erythematosus with positive or unknown antiphospholipid antibodies; unexplained vaginal bleeding; current or history of breast cancer; acute liver disease or cirrhosis; benign or malignant tumor of the liver; use of rifampicin, and/or anticonvulsants (barbiturates, phenytoin, phenobarbital, carbamazepine, oxcarbazepine, primidone, topiramate), and/or herbal products containing St. John’s Wort (Hypericum perforatum); > 1 sexual partner in the last 3 months; diagnosis or treatment for sexually transmitted infection (STI) within past 30 days for her or her partner (excluding recurrent genital herpes or condyloma); known HIV positive status for her or her partner; any condition (social or medical) which in the opinion of the Investigator would make study participation unsafe, would interfere with adherence to study requirements or complicate data interpretation; and BMI > 30kg/m^2^ (excluded only from the pharmacokinetic (PK) subgroup).

*3.3 Pharmacokinetics*

*3.3.1* *Total LNG Concentrations During up to Forty-Eight Months of Use*

Among N=3769 specimens collected at month 1, 6, 12, 18, 24, 30, 36, 42, or 48 visits, 156 (4.1%) were excluded, with no difference between groups (p=1.0). Specific (possibly overlapping) reasons for specimens being excluded include: 107 (2.8%) out of visit window; 16 (0.4%) duplicate results in the same visit window; 13 (0.3%) obtained when a participant reported using a medication that can impact metabolism of LNG (six anticonvulsants, five antitubercular agents, and two hormonal contraceptives); 10 (0.3%) with ethinyl estradiol (EE) detected in the specimen. and nine (0.2%) gross outliers (≥3000 pg/mL on or after month one).

Total plasma LNG concentrations in the PK population uniformly exceeded 200 pg/mL within 24 hours of implant insertion in both groups. The mean C_max_ in the Sino-implant (II) and Jadelle® group, respectively, was 833 and 962 pg/mL; mean T_max_ was 5.4 and 4.3 days; and mean AUC_0-6m_ was 2489 and 2862 pg∙months/mL. Given this was not a traditional bioavailability trial and had less frequent sampling, the C_max_ and T_max_ may be somewhat imprecise estimates.

In the Sino-implant (II) group, mean concentrations decreased from 428 pg/mL one month after insertion to 310, 252, 220, and 205 pg/mL at months 12, 24, 36, and 48, respectively (Figure 2). In the Jadelle® group, mean concentrations generally decreased from 453 pg/mL at month one to 314, 310, 276, and 299 pg/mL at months 12, 24, 36, and 48, respectively. The observed trend in decreasing geometric mean ratios (GMRs) over time was significant (p<0.001) in an exploratory test of no difference in log-linear slopes A significantly higher percentage of Sino-implant (II) participants had total LNG concentrations below 200 pg/mL at one or more time points during the first 4 years of follow-up (288 (56.0%) Sino-implant (II) versus 47 (34.6% of Jadelle users – p<0.01). Likewise, forty-three (8.4%) Sino-implant (II) users had total LNG concentrations below 100 pg/mL at one or more time points during follow-up (none before month 18), compared to one (0.7%) Jadelle® user (p<0.001).

*3.3.2 SHBG and Free LNG Index (FLI) During up to Forty-Eight Months of Use*

There was a rapid reduction in SHBG levels immediately following insertion of either implant type in the PK population: from a mean of 91.5 nmol/L at baseline to a minimum of 33.9 nmol/L at month 1 for Sino-implant (II), and from 86.7 to 32.1 nmol/L at month 3 for Jadelle®. Combined with rising total LNG concentrations, there was a corresponding rapid increase in the FLI, which achieved a maximum mean of 5.2 at day 7 for Sino-implant (II) and 5.1 at month 1 for Jadelle®.

SHBG levels and FLI values remained comparable between groups in the first 90 days of implant use but diverged markedly thereafter, with significantly less SHBG suppression (GMR=1.26; p=0.02) and lower FLI values (GMR=0.73; p<0.001) by month 6 of Sino-implant (II) use in the As-Treated Population (Supplementary Figure 1). SHBG levels rebounded somewhat after month 24 in both groups, but there was on average less SHBG suppression and significantly lower FLI values through month 48 (GMR FLI=0.59; p<0.001) in the Sino-implant (II) group.

**Supplementary Figure 1** Arithmetic mean sex hormone binding globulin (SHBG) (diamonds; left axis) and free levonorgestrel index (FLI) (circles; right axis) during 48 months of use in women randomized to Sino-implant (II) or Jadelle® insertion (95% CIs are shifted slightly for visibility).


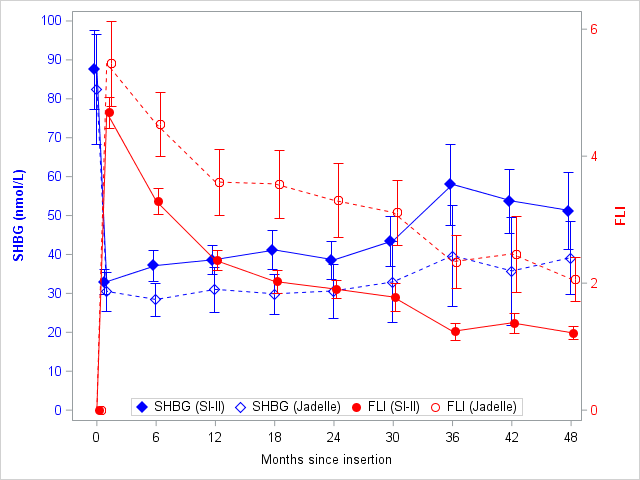

Supplement: Supplementary file 1 — Supplementary material [file mmc1.docx]
